# Disparities in Monoclonal Antibody (mAb) Treatment Usage in the Military Health System During the COVID-19 Pandemic

**DOI:** 10.1007/s11606-025-09715-z

**Published:** 2025-07-15

**Authors:** Elta Liang, Kevin Chuang, Cathaleen Madsen, Kevin K. Chung, Christian L. Coles, Tracey Pérez Koehlmoos

**Affiliations:** 1 The Henry M. Jackson Foundation for the Advancement of Military Medicine, Inc., Bethesda, MD USA; 2 Uniformed Services University of the Health Sciences, Bethesda, MD USA; 3 SeaStar Medical, Denver, CO USA

## Abstract

**Background:**

Monoclonal antibodies (mAbs) were identified in 2020 as potential curative agents for COVID-19, particularly in high-risk populations. However, studies report differences in receipt of mAbs by race, sex, and insurance status. We hypothesized that Military Health System (MHS) beneficiaries, who are universally insured, would have approximately equal uptake of mAbs across sociodemographic categories.

**Methods:**

Beneficiaries (active-duty service members, dependents, retirees) aged 12–64, receiving a COVID-19 diagnosis and outpatient treatment between November 9, 2020, and January 24, 2022, were identified from claims data in the MHS Data Repository (MDR). Analyses comprised descriptive statistics on demographics and frequency of COVID-19 mAb treatment for all eligible beneficiaries and by sex, race, sponsor rank, and risk status. Unadjusted and adjusted odds ratios (OR) for likelihood of receiving mAb treatment were obtained using multivariable logistic regression models for all beneficiaries as well as only those classified as “high risk.”

**Results:**

Of 221,036 COVID-19 patients receiving outpatient care, 9907 (4.5%) received mAb treatment. Beneficiaries who received treatment were predominately White (53%), female (57%), ages 45–64 (64%), dependents (56%), associated with enlisted rank (77%), and not at high risk of developing severe COVID-19 and/or hospitalization (86%). Greater uptake of mAbs by American Indian/Alaska Native and lesser uptake by Black and Asian compared to White patients, and by female compared to male patients, concurs with published results in the greater US population.

**Conclusions:**

Despite universal insurance, significant differences were observed in receipt of mAbs by race, rank (proxy for socioeconomic status), and sex. These results suggest that factors beyond insurance play roles in determining who receives mAbs. The provision of mAbs to non-high-risk patients also suggests a role for either patient- or provider-induced demand. Further research is needed to determine the scope of factors affecting mAb uptake in the MHS, and in other large health systems.

**Supplementary Information:**

The online version contains supplementary material available at 10.1007/s11606-025-09715-z.

## BACKGROUND

Between 2020 and 2022, there were nearly 100,000,000 infections and approximately 1,000,000 deaths attributed to SARS-CoV-2 in the USA.^1,2^ The development of therapies to prevent severe illness from COVID-19 in high-risk populations became crucial during the early pandemic. Researchers quickly identified monoclonal antibodies (mAbs) as agents that could potentially reduce severe morbidity and mortality from COVID-19.^3^ Monoclonal antibodies are laboratory-produced antibodies that target a specific protein on a virus to prevent it from entering or duplicating in human cells.^4^ This mechanism mimics the immune response and assists the body in fighting infections. Therefore, mAbs can be effective in both the prevention and treatment of COVID-19 infection.^4^

Between November 2020 and February 2021, three mAbs, bamlanivimab, REGEN-CoV (casirivimab-imdevimab), and bamlanivimab-etesevimab, were given emergency use authorization by the Food and Drug Administration (FDA) for the treatment of mild to moderate illness from COVID-19 in adults and pediatric patients who were at high risk for progression to severe COVID-19, hospitalization, or death.^5^ Despite efforts from the government and healthcare systems to promote equitable distribution of mAbs to disadvantaged populations, data indicates lower mAb uptake among these groups.^6^ Multiple reviews of healthcare system data within the USA found that White patients were more likely than Blacks and other racial minority groups to receive mAb treatment.^6,7^ Moreover, high-risk patients without health insurance and patients living in high social vulnerability zip codes were less likely to receive mAbs.^6,8,9^ Although males were more likely to be hospitalized and admitted to the ICU for COVID-19 infections, national studies found higher uptake of mAbs in females.^7,10^

To our knowledge, no study has investigated whether the disparities in mAb usage identified across the USA also extend to the Military Health System (MHS). The MHS delivers care to approximately 9.6 million beneficiaries, including both active and non-active-duty service members, dependents, and retirees.^11^ Unlike the civilian healthcare system, the MHS offers universal insurance coverage to all beneficiaries through TRICARE, typically at no cost to the patient.^12^ Given that universal insurance has often been linked to increased access to care and reduced health disparities, the MHS may be a valuable model for examining whether there was equitable distribution of mAbs during the COVID-19 pandemic.^13^ In this context, we examined whether there were disparities in mAb usage within the MHS population by race, sex, and socioeconomic status. We hypothesized that the uptake of mAbs across each demographic category would be similar due to the MHS’s provision of universal coverage to all beneficiaries.

## METHODS

### Study Design, Population, and Data Source

This retrospective study identified TRICARE Prime and Plus beneficiaries ages 12–64 from November 9, 2020, to January 24, 2022, using claims data from the Military Health System Data Repository (MDR). Beneficiaries were eligible if they received a COVID-19 diagnosis and were seen within outpatient care during the study period. We excluded beneficiaries ages 65 and older due to the loss in transparency of care when Medicare becomes the primary insurance payer. Additionally, due to intermittent access to the MHS, National Guard and Reserves, along with their dependents, were excluded.

Clinical data from the MDR along with *International Classification of Disease, 10th Edition* (ICD-10) codes were used to identify COVID-19 diagnoses. This study looked at three monoclonal antibody (mAb) treatments: bamlanivimab, casirivimab-imdevimab (REGEN-COV), and bamlanivimab-etesevimab. Treatments were identified using Current Procedural Terminology/Healthcare Common Procedure Coding System (CPT/HCPCS) codes. Based on each mAb manufacturer’s guidelines, ICD-10 codes along with pharmaceutical data from the MDR were utilized to determine if beneficiaries were at high risk of developing severe COVID-19 and/or being hospitalized. All CPT/HCPCS and ICD-10 codes can be found in Table S1.^14–17^ We employed the beneficiary’s Defense Enrollment Eligibility Reporting System (DEERS) record to obtain demographic information such as categorical age, biological sex, beneficiary status (active duty, dependent, and retiree), self-reported race (White, Black, Asian/Pacific Islander, American Indian/Alaska Native), sponsor rank (junior enlisted, senior enlisted, junior officer, senior officer, warrant officer), associated branch of service (Army, Air Force, Navy, and Marine Corps), and healthcare setting (direct care and private care) for analysis. Sponsor rank was determined to be a suitable proxy for socioeconomic status as shown in prior studies of TRICARE beneficiaries, with enlisted rank being a correlate of lower socioeconomic status.^18,19^ Ranks of dependents and retirees are based upon their sponsor’s rank and rank prior to retirement, respectively. Beneficiaries associated with the US Space Force were grouped with those in the US Air Force due to their small sample size. As a result of high rates of missing data, ethnicity (Hispanic or non-Hispanic) was not retained for analysis.

### Statistical Analysis

Analyses comprised descriptive statistics on patient demographics and frequency of COVID-19 mAb treatment for all eligible beneficiaries and by sex, race, sponsor rank, and risk status. Unadjusted and adjusted odds ratios (OR) for patient likelihood of receiving mAb treatment were obtained using simple and multivariable logistic regression models for all beneficiaries as well as only those classified as “high risk.” Multivariable models included sex, race, sponsor rank, and risk status as independent predictors for receipt of mAb treatment with beneficiary status and categorical age as potential confounders. To address the high rates of missing race data, we applied the reweighted estimating equations (RWEE) imputation technique to the logistic regression models.^20^ Observed and weighted frequencies for race are provided in Table S2.

Statistical significance of odds ratio (OR) and 95% confidence interval (CI) were determined, a priori, as exclusive of 1.00 and *α* = 0.05. This study was reviewed and found exempt by the Institutional Review Board at the Uniformed Services University of the Health Sciences. All statistical analyses were conducted using SAS 9.4 (SAS Institute Inc). We adhered to the Strengthening the Reporting of Observational Studies in Epidemiology (STROBE) guidelines.

## RESULTS

**Figure 1:**
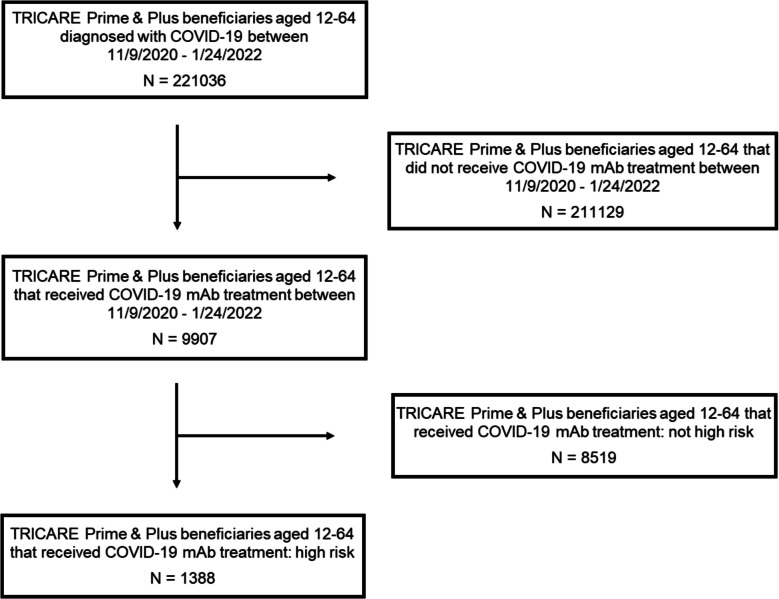
CONSORT diagram. Abbreviations: mAb, monoclonal antibody.

We identified 221,036 beneficiaries with a COVID-19 diagnosis receiving outpatient care during the study period, of which 9907 (4.5%) received mAb treatment (Fig. 1). Beneficiaries who received treatment were predominately female (57%), of White race (53%), ages 45–64 (64%), dependents (56%), in an enlisted rank (77%), and not at high risk of developing severe COVID-19 and/or hospitalization (86%) (Table 1).

**Table 1:** Demographics of Eligible Beneficiaries by Receipt of COVID-19 Monoclonal Antibody Treatment

Characteristic	Total diagnosed with COVID-19 *n* (% of total)	Did not receive treatment *n* (% of total)	Received treatment		
			**Total treated** ***n* (% of total)**	**High risk** ***n* (% of total)**	**Not high risk** ***n* (% of total)**
Total	221,036	211,129	9907	1388	8519
Age (years)					
12–17	25,741 (11.6%)	25,524 (12.1%)	217 (2.2%)	17 (1.2%)	200 (2.4%)
18–24	62,622 (28.3%)	62,187 (29.4%)	435 (4.4%)	48 (3.5%)	387 (4.5%)
25–34	62,951 (28.5%)	61,912 (29.3%)	1039 (10.5%)	135 (9.7%)	904 (10.6%)
35–44	32,894 (14.9%)	30,963 (14.7%)	1931 (19.5%)	259 (18.7%)	1672 (19.6%)
45–54	19,870 (9.0%)	17,259 (8.2%)	2611 (26.4%)	322 (23.2%)	2289 (26.9%)
55–64	16,958 (7.7%)	13,284 (6.3%)	3674 (37.1%)	607 (43.7%)	3067 (36.0%)
Sex					
Female	92,753 (42.0%)	87,136 (41.3%)	5617 (56.7%)	741 (53.4%)	4876 (57.2%)
Male	128,283 (58.0%)	123,993 (58.7%)	4290 (43.3%)	647 (46.6%)	3643 (42.8%)
Race*					
White	116,387 (52.7%)	111,160 (52.7%)	5227 (52.8%)	738 (53.2%)	4489 (52.7%)
Black	29,096 (13.2%)	28,159 (13.3%)	937 (9.5%)	167 (12.0%)	770 (9.0%)
Asian/Pacific Islander	9991 (4.5%)	9704 (4.6%)	287 (2.9%)	41 (2.9%)	246 (2.9%)
American Indian/Alaska Native	1640 (0.7%)	1545 (0.7%)	95 (1.0%)	14 (1.0%)	81 (1.0%)
Other	23,217 (10.5%)	22,490 (10.7%)	727(7.3%)	98 (7.1%)	629 (7.4%)
Missing	40,705 (18.4%)	38,071 (18.0%)	2634 (26.6%)	330 (23.8%)	2304 (27.0%)
Beneficiary status					
Active duty	104,117 (47.1%)	103,266 (48.9%)	851 (8.6%)	142 (10.2%)	709 (8.3%)
Dependent	94,410 (42.7%)	88,859 (42.1%)	5551 (56.0%)	728 (52.5%)	4823 (56.6%)
Retiree	22,509 (10.2%)	19,004 (9.0%)	3505 (33.4%)	518 (37.3%)	2987 (35.1%)
Branch					
Army	83,830 (37.9%)	79,889 (37.8%)	3941 (39.8%)	655 (47.2%)	3286 (38.6%)
Air Force/Space Force	67,140 (30.4%)	63,954 (30.3%)	3186 (32.2%)	390 (28.1%)	2796 (32.8%)
Navy	43,123 (19.5%)	41,377 (19.6%)	1746 (17.6%)	231 (16.6%)	1515 (17.8%)
Marine Corps	20,435 (9.3%)	19,740 (9.4%)	695 (7.0%)	84 (6.1%)	611 (7.2%)
Other	6508 (2.9%)	6169 (2.9%)	339 (3.4%)	28 (2.0%)	311 (3.6%)
Rank					
Junior enlisted	50,215 (22.7%)	49,633 (23.5%)	582 (5.9%)	91 (6.6%)	491 (5.8%)
Senior enlisted	106,676 (48.3%)	99,619 (47.2%)	7057 (71.2%)	1011 (72.8%)	6046 (71.0%)
Junior officer	19,829 (9.0%)	19,052 (9.0%)	777 (7.8%)	95 (6.8%)	682 (8.0%)
Senior officer	11,519 (5.2%)	10,712 (5.1%)	807 (8.2%)	104 (7.5%)	703 (8.2%)
Warrant officer	4545 (2.0%)	4201 (2.0%)	344 (3.5%)	48 (3.5%)	296 (3.5%)
Other^†^	28,252 (12.8%)	27,912 (13.2%)	340 (3.4%)	39 (2.8%)	301 (3.5%)
Risk status					
High risk	10,032 (4.5)	8644 (4.1)	1388 (14.0)	N/A	N/A
Not high risk	211,004 (95.5)	202,485 (95.9)	8519 (86.0)	N/A	N/A
Sector					
Direct	116,384 (52.7%)	115,355 (54.6%)	1029 (10.4%)	274 (19.7%)	755 (8.9%)
Private	104,652 (47.3%)	95,774 (45.4%)	8878 (89.6%)	1114 (80.3%)	7764 (91.1%)

^*^In the Military Health System Data Repository, race does not include ethnicity. Therefore, categories reported are race only. Additionally, race is self-reported and Military Health System beneficiaries could select the Other race value to identify as multiracial. Details regarding individual races for Other race are not available or reported

^†^Other sponsor rank includes cadets and individuals eligible for care, but who do not hold a military rank

From the adjusted analysis, female beneficiaries were more likely to receive mAb treatment (OR, 1.16; 95% CI, 1.08–1.26) compared to male beneficiaries (Table 2). In comparison to White beneficiaries, Black beneficiaries and Asian/Pacific Islander beneficiaries were less likely to receive mAb treatment (OR, 0.75; 95% CI, 0.70–0.81 and OR, 0.58; 95% CI, 0.51–0.65 respectively). For sponsor rank, both junior and senior enlisted (OR, 1.37; 95% CI, 1.19–1.57 and OR, 1.26; 95% CI, 1.15–1.37 respectively) along with warrant officers (OR, 1.23; 95% CI, 1.06–1.44) had a higher likelihood of receiving mAb treatment. Adjusted analysis also showed that beneficiaries classified as high risk and those receiving private sector care were more likely to receive mAb treatment (OR, 1.30; 95% CI, 1.21–1.40 and OR, 4.46; 95% CI, 4.06–4.90 respectively) compared to individuals who were not at high risk and those receiving direct care respectively.

**Table 2:** Logistic Regression Results for Receipt of COVID-19 Monoclonal Antibody Treatment

Variable	Unadjusted OR (95% CI) *N* = 180331	Adjusted OR (95% CI) *N* = 180331
Sex		
Female	1.76 (1.68–1.84)^†^	1.16 (1.08–1.26)^†^
Male	1 (reference)	1 (reference)
Race^‡^		
White	1 (reference)	1 (reference)
Black	0.70 (0.66–0.75)^†^	0.75 (0.70–0.81)^†^
Asian/Pacific Islander	0.63 (0.56–0.71)^†^	0.58 (0.51–0.65)^†^
American Indian/Alaska Native	1.31 (1.07–1.60)^†^	1.24 (0.99–1.54)
Other	0.69 (0.64–0.74)^†^	0.80 (0.75–0.89)^†^
Rank		
Junior enlisted	0.14 (0.13–0.16)^†^	1.37 (1.19–1.57)^†^
Senior enlisted	0.92 (0.85–1.01)	1.26 (1.15–1.37)^†^
Junior officer	0.51 (0.45–0.57)^†^	1.09 (0.96–1.23)
Senior officer	1 (reference)	1 (reference)
Warrant officer	1.10 (0.95–1.27)	1.23 (1.06–1.44)^†^
Other^§^	0.18 (0.15–0.20)^†^	0.90 (0.76–1.06)
Risk status		
High risk	4.11 (3.84–4.39)^†^	1.30 (1.21–1.40)^†^
Not high risk	1 (reference)	1 (reference)
Sector		
Direct	1 (reference)	1 (reference)
Private	9.56 (8.92–10.26)^†^	4.46 (4.06–4.90)^†^

Abbreviations: *OR*, odds ratio; *CI*, confidence interval

Multivariable logistic regression models were adjusted by categorical age and beneficiary status

^†^Indicates statistical significance with *p* < 0.05

^‡^Race is self-reported and Military Health System beneficiaries could select the Other race value to identify as multiracial. Details regarding individual races for Other race are not available or reported

^§^Other sponsor rank includes cadets and individuals who are eligible for care but do not hold a military rank

When evaluating the adjusted analysis of only those classified as “high risk,” we observed that Black beneficiaries and Asian/Pacific Islander beneficiaries had a lower likelihood of receiving mAb treatment (OR, 0.74; 95% CI, 0.62–0.89 and OR, 0.47; 95% CI, 0.34–0.65 respectively) compared to White beneficiaries (Table 3).

**Table 3:** Logistic Regression Results for Receipt of COVID-19 Monoclonal Antibody Treatment among High-Risk Beneficiaries

Variable	Unadjusted OR (95% CI) *N* = 7760	Adjusted OR (95% CI) *N* = 7760
Sex		
Female	1.31 (1.15–1.48)^†^	1.09 (0.88–1.36)
Male	1 (reference)	1 (reference)
Race^‡^		
White	1 (reference)	1 (reference)
Black	0.73 (0.61–0.87)^†^	0.74 (0.62–0.89)^†^
Asian/Pacific Islander	0.53 (0.39–0.74)^†^	0.47 (0.34–0.65)^†^
American Indian/Alaska Native	1.23 (0.72–2.12)	1.14 (0.66–1.98)
Other	0.66 (0.54–0.81)^†^	0.67 (0.54–0.83)^†^
Rank		
Junior enlisted	0.45 (0.32–0.63)^†^	0.96 (0.66–1.39)
Senior enlisted	0.77 (0.60–0.98)^†^	0.82 (0.64–1.05)
Junior officer	0.70 (0.50–0.98)^†^	0.81 (0.57–1.14)
Senior officer	1 (reference)	1 (reference)
Warrant officer	1.15 (0.77–1.73)	1.10 (0.73–1.67)
Other^§^	0.22 (0.14–0.33)^†^	0.20 (0.13–0.31)^†^
Sector		
Direct	1 (reference)	1 (reference)
Private	1.77 (1.52–2.06)^†^	1.05 (0.88–1.26)

Abbreviations: *OR*, odds ratio; *CI*, confidence interval

Multivariable logistic regression models were adjusted by categorical age and beneficiary status

^†^Indicates statistical significance with *p* < 0.05

^‡^Race is self-reported and Military Health System beneficiaries could select the Other race value to identify as multiracial; however, details regarding individual races for Other race are not available or reported

^§^Other sponsor rank includes cadets and individuals eligible for care, but who do not hold a military rank

## DISCUSSION

Through this investigation, we sought to assess whether disparities in usage of mAb treatment for COVID-19 existed within the Military Health System between the period of November 2020 and January 2022. After analyzing medical claims data for three prominent mAbs, it was found that 4.5% of TRICARE beneficiaries who were diagnosed with COVID-19 during the study period received mAb treatment. Beneficiaries who were at high risk for severe illness from COVID-19 were more likely to receive mAbs than those who were not at high risk. Despite universal healthcare coverage, disparities in the likelihood of receiving mAbs were observed by race, sex, and sponsor rank, as well as between private and direct care sectors of the MHS.

Notably, our results showed that, in comparison to White beneficiaries, Black and Asian/Pacific Islander beneficiaries were less likely to receive mAbs. This finding is consistent with research conducted within the civilian population that has shown lower uptake of mAbs among certain minority groups. For example, a review of data from the National Patient-Centered Clinical Research Network reported that Black and Asian patients were significantly less likely to receive mAbs than White patients, with a 22% and 48% gap in usage, respectively.^7^ Additionally, a review of electronic health records from four health systems in the USA found that Black patients were less likely to be treated with mAbs than White patients.^6^

It has been proposed that a complex interplay of social, cultural, and clinical factors may be associated with rates of acceptance for mAbs among different racial/ethnic groups.^21^ For example, Bierle et al. found that Black and Asian patients, and those who identified as having a primary language that was not English, were more likely to refuse mAb therapy.^21^ Factors such as health system mistrust, medical misinformation, and differences in cultural beliefs surrounding medicine that have also been associated with COVID-19 vaccine hesitancy may factor into decisions to accept mAb therapy.^22^

Despite adjustment for potential covariates, our study revealed that female beneficiaries had a higher likelihood of receiving mAbs in comparison to male beneficiaries. Although this finding aligns with existing studies based on national healthcare system data, which demonstrate slightly higher percentages of mAb uptake by female patients,^7,8,10^ we hypothesize that our finding is driven by the different arenas in which beneficiaries sought care. Current MHS guidelines prioritize direct care for active-duty service members^23^, which in 2021 were ~ 83% male.^24^ Additional factors which may drive this difference have been observed in the greater US population, and include the greater likelihood of females to seek medical attention than males.^25,26^ For example, a cross-sectional study of adults with confirmed COVID-19 diagnoses in the USA found that females had higher odds of seeking treatment from their physician in comparison to males.^27^ Alternatively, there could have been implicit biases or systemic issues that led to differences in treatment allocation by sex.^28^ Nonetheless, our findings are concerning considering the evidence that males with COVID-19 are at higher risk for severe illness, hospitalization, and death, in comparison to females.^27,29^ These findings are also concerning from a military perspective, as the Armed Forces are majority male and must be fit for duty in order to carry out their mission. Further research is needed to determine the reasons influencing these decisions, particularly for active-duty service members.

One significant finding was the provision of mAbs to patients who were not at high risk from COVID-19 infection. In direct care, 755 of 1029 patients (73.4%) and in purchased care, 7,764 of 8878 patients (87.5%) who received mAbs did not meet the clinical criteria. There are several possible explanations. One is incomplete coding which fails to capture the full scope of patient risk factors; however, it seems unlikely that over 70% of patients in both care sectors were miscoded. A second is social determinants of health which are not normally recorded in a healthcare setting, such as the need to care for family members, or low food security which is independently correlated with COVID-19 infection.^30^ It is notable that military populations may also experience food insecurity, particularly in the enlisted ranks.^31^ In our study, enlisted personnel and their beneficiaries comprised approximately 71% of those diagnosed with COVID and 77% of those receiving mAbs, with a slightly higher percentage of those at high risk than not. However, social determinants could not be conclusively identified from our dataset. A third is older age, as those aged 55 + accounted for only 7.7% of those diagnosed with COVID, but 37.1% of those receiving mAbs. This is also the only group where the number of patients receiving mAbs were at high risk vs. not at high risk, and therefore mAb provision in this group may reflect an abundance of caution on the part of providers. Neither our dataset nor the existing literature provides a ready explanation for the use of mAbs outside of high-risk groups, and therefore more research is needed to answer this question.

### Strengths and Limitations

This study has several strengths. It constitutes a population-level examination of prescribing practices across over 200,000 patients from multiple socioeconomic and demographic backgrounds. Because this population is nationally representative and predominantly working age, the study is not limited to those of lower income or advanced age, such as studies in the Medicaid and Medicare populations, respectively. As MHS beneficiaries have universal insurance and generally at least one fully employed family member, the results of this study can be used to assess the disparities which persist despite insurance and job status. This study also has several limitations. As a secondary data analysis, it is subject to potential error, including missing data or inaccurate coding, and does not capture clinical nuances that may appear in provider notes, including clinical findings and patient vital signs. Our analysis did not account for any changes in the eligibility criteria for high risk during the study period. This study also does not capture care provided outside the MHS. As race was imputed for non-active-duty beneficiaries, this study may not have captured a full picture of disparities. Finally, this study did not stratify by multiple factors, such as age with rank or gender with race, which limits the granularity of the findings. Further research is needed to answer this question.

## CONCLUSION

Despite the provision of universal insurance to MHS beneficiaries, this study showed differences in mAb treatment by race, rank, sex, and care arena. Findings by race and sex are in line with studies in the greater US population, suggesting that factors beyond insurance are affecting the provision of mAbs. While those at high risk of severe COVID-19 and associated complications were more likely to receive mAbs, the greatest number of beneficiaries receiving mAbs were not at high risk. The reasons for this were not determinable from our dataset nor from existing literature, and therefore further research is needed to answer this question.

## Supplementary Information

Below is the link to the electronic supplementary material.

Supplementary file1 (DOCX 14 KB)

## Data Availability

The data that support the findings of this study are available from the United States Defense Health Agency. Restrictions apply to the availability of these data, which were used under Federal Data User Agreements for the current study, and so are not publicly available.

## Declarations

### Disclaimer

The contents of this publication are the sole responsibility of the authors and do not necessarily reflect the views, assertions, opinions, or policies of the Uniformed Services University of the Health Sciences (USUHS); the Henry M. Jackson Foundation for the Advancement of Military Medicine, Inc. (HJF); the Department of Defense (DoD); the Departments of the Army, Navy, or Air Force; or SeaStar Medical. Mention of trade names, commercial products, or organizations does not imply endorsement by the US Government.
