# Supplementary material for: Disparities in Monoclonal Antibody (mAb) Treatment Usage in the Military Health System During the COVID-19 Pandemic
Source: J Gen Intern Med. 2025 Jul 15;40(14):3444–51. doi: 10.1007/s11606-025-09715-z (PMC12586743; doi:10.1007/s11606-025-09715-z)
Supplement: Supplementary file 1 — Supplementary file1 (DOCX 14 KB) [file 11606_2025_9715_MOESM1_ESM.docx]

| **Table S1. Codes for Identifying High Risk Conditions (ICD-10) and Monoclonal Antibody Treatments (CPT/HCPCS)** | |
| --- | --- |
| **Condition/Treatment** | **Code** |
| COVID-19 | U07.1 |
| Bamlanivimab | Q0239, M0239 |
| Casirivimab & Imdevimab  (REGEN-COV) | Q0243, Q0244, M0243, M0244 |
| Bamlanivimab & Etesevimab | Q0245, M0245 |
| Body Mass Index > 25 | Z68.26, Z68.27, Z68.28, Z68.29, Z68.30, Z68.31, Z68.32, Z68.33, Z68.34, Z68.35, Z68.36, Z68.37, Z68.38, Z68.39, Z68.4, E66.0, E66.1, E66.2, E66.8 |
| Body Mass Index ≥ 85th Percentile | Z68.53, Z68.54 |
| Chronic Kidney Disease | N18 |
| Diabetes | E10, E11 |
| Cardiovascular Disease | I43, I50, I09.9, I11.0, I13.0, I13.2, I25.5, I42.0, I42.5, I42.6, I42.7, I42.8, I42.9, P29.0 |
| Congenital Heart Disease | Q20, Q21, Q22, Q23, Q24, Q25, Q26, Q27, Q28 |
| Hypertension | I10, I11, I12, I15 |
| Chronic Obstructive Respiratory Disease | I27.8, I27.9, J40, J41, J42, J43, J44, J45, J46, J47, J60, J61, J62, J63, J64, J65, J66, J67, J68.4, J70.1, J70.3 |
| Other Chronic Respiratory Disease | I26, I27, I28.0, I28.8, I28.9 |
| Sickle Cell Disease | D57.20 |
| Pregnancy | Z33.1 |
| Neurodevelopmental Disease | G10, G11, G12, G13, G14, G20, G21, G23, G24, G25, G26, G30, G31, G32, G35, G36, G37, G40, G43, G44, G45, G46, G47, G50, G51, G52, G53, G54, G55, G56, G57, G58, G59, G60, G61, G62, G63, G64, G65, G70, G71, G72, G73, G89, G90 G91, G92, G93, G94, G95, G96, G97, G98, G99 |
| Immunosuppressive Disease | B01, B57, B95, D51, D53, D55, D56, D57, D58, D59, D60, D61, D64, D69, D70, D72, D80, D81, D82, D83, D86, E05, E07, E10, E31, E85, G04, G05, G13, G31, G32, G35, G36, G37, G56, G57, G58, G59, G60, G61, G62, G63, G64, G65, G90, H15, H16, H18, H20, H21, H30, H35, H44, H46, H49, H83, I00, I01, I24, I33, I73, I77, K20, K50, K51, K68, K73, K74, K86, L10, L11, L30, L40, L43, L50, L52, L63, L80, L88, L93, L94, L95, L98, M02, M05, M06, M08, M27, M30, M31, M32, M33, M34, M35, M36, M45, M60, M79, M86, M89, N00, N01, N02, N03, N04, N05, N06, N07, N08, N28, N45, N70, N80, A18.4, A69.2, B01.1, B33.2, D69.0, D84.1, D89.1, E06.3, E27.1, G31.2, G36.0, G37.5, G47.4, G61.0, G70.0, G70.8, G73.1, H10.9, H16.0, H81.0, I40.1, I51.4, I67.7, I77.6, J39.2, K22.0, K75.4, K90.0, L12.0, L12.1, L12.2, L13.0, L28.0, L30.9, L41.0, L73.2, L90.0, L93.0, M05.2, M06.1, M08.1, M12.3, M30.1, M30.3, M31.0, M31.3, M31.4, M31.6, M31.7, M32.8, M35.0, M35.2, M35.4, M61.0, M61.1, M79.7, M94.1, N02.8, N30.1, N48.0, N90.4, O26.4, P00.8, Q24.6, Q39.5, T78.3 |
| Treatment for Immunosuppressive Disease | CYCLOSPORINE, GENGRAF, NEORAL, SANDIMMUNE, TACROLIMUS, ASTAGRAF XL, ENVARSUS XR, PROGRAF, SIROLIMUS, RAPAMUNE, EVEROLIMUS, AFINITOR, ZORTRESS, MYCOPHENOLATE, CELLCEPT, MYFORTIC, MYHIBBIN, METHOTREXATE, RHEUMATREX, TREXALL, AZATHIOPRINE, AZASAN, IMURAN, CYCLOPHOSPHAMIDE, CYTOXAN, LEFLUNOMIDE, ARAVA, ANAKINRA, KINERET, ABATACEPT, ORENCIA, ETANERCEPT, ENBREL, ERELZI, ETICOVO, ADALIMUMAB, ABRILADA, AMJEVITA, CYLTEZO, HADLIMA, HULIO, HUMIRA, HYRIMOZ, IDACIO, SIMLANDI, YUFLYMA, YUSIMRY, INFLIXIMAB-ABDA, RENFLEXIS, INFLIXIMAB-DYYB, INFLECTRA, INFLIXIMAB, REMICADE, SULFASALAZINE, AZULFIDINE, HYDROXYCHLOROQUINE, PLAQUENIL, CERTOLIZUMAB, CIMZIA, GOLIMUMAB, SIMPONI, TOCILIZUMAB, ACTEMRA, TOFIDENCE, TYENNE |
| Tracheotomy | Z93.0 |
| Gastrotomy | Z93.1 |
| Abbreviations: ICD-10, International Classification of Disease 10th Edition; CPT/HCPCS, Current Procedural Terminology/Healthcare Common Procedure Coding System | |
